# A genetic and virulence characterization of Brazilian strains of *Mycoplasma hyopneumoniae*

**DOI:** 10.3389/fmicb.2023.1280588

**Published:** 2023-11-22

**Authors:** Leonardo Teófilo Toledo, Luiz Fernando Lino de Souza, Carlos Eduardo Real Pereira, Richard Costa Polveiro, Gustavo Costa Bressan, Ricardo Seiti Yamatogi, Kwangcheol Casey Jeong, Fernanda Simone Marks, Caio Augustus Diamantino, Victor Hugo Rabelo de Carvalho, Clarisse Sena Malcher, Fernando Antônio Moreira Petri, Luis Guilherme de Oliveira, Maria Aparecida Scatamburlo Moreira, Abelardo Silva-Júnior

**Affiliations:** ^1^Department of Veterinary Medicine, Federal University of Viçosa, Viçosa, Brazil; ^2^Department of Biochemistry and Molecular Biology, Federal University of Viçosa, Viçosa, Brazil; ^3^Emerging Pathogens Institute, University of Florida, Gainesville, FL, United States; ^4^School of Agricultural and Veterinarian Sciences, São Paulo State University (Unesp), São Paul, Brazil; ^5^Institute of Biological and Health Sciences, Federal University of Alagoas, Maceió, Brazil

**Keywords:** UFV01, UFV02, respiratory disease, experimental challenge, Swine. *Mycoplasma hyopneumoniae*, genome, virulence, lesion

## Abstract

*Mycoplasma hyopneumoniae* (*M. hyopneumoniae*) is considered the primary causative agent of porcine enzootic pneumonia (EP), a chronic contagious respiratory disease that causes economic losses. Obtaining new pathogenic isolates and studying the genome and virulence factors are necessary. This study performed a complete sequencing analysis of two Brazilian strains, UFV01 and UFV02, aiming to characterize the isolates in terms of the virulence factors and sequence type. The complete genome analysis revealed the main virulence genes (*mhp385*, *mhp271*, *MHP_RS03455*, *p102*, *p97*, *p216*, *MHP_RS00555*, *mhp107*) and ST-123, the presence of three toxin-related genes (*tlyC*, *PLDc_2* and *hcnC*), and some genetic groups specific to these two isolates. Subsequently, the pathogenicity of the isolates was evaluated via an experimental infection conducted in a swine model. The study was divided into three groups, namely a negative control group (*n* = 4) and two test groups (*n* = 8), totaling 20 animals. They were challenged at 35 days of age with 10^7^ CCU (Color Changing Units) *M. hyopneumoniae* via the intratracheal route. The UFV01 group showed earlier and higher seroconversion (IgG) (100%), while only 50% of the UFV02 group seroconverted. The same trend was observed when analyzing the presence of IgA in the bronchoalveolar lavage fluid (BALF) at 35 days post-infection (dpi). The UFV01 group had a mean macroscopic lesion score of 11.75% at 35 dpi, while UFV02 had 3.125%. Microscopic lesions were more severe in the UFV01 group. Based on laryngeal swab samples evaluated by qPCR, and the detection began at 14 days. The UFV01 group showed 75% positivity at 14 dpi. The UFV02 group also started excreting at 14 dpi, with a positivity rate of 37.5%. The results indicate that the UFV01 isolate exhibits higher virulence than UFV02. These findings may aid in developing new vaccines and diagnostic kits and establishing experimental models for testing.

## 1 Introduction

*Mycoplasma hyopneumoniae* (*M. hyopneumoniae*) is the primary agent of enzootic pneumonia (EP), belonging to the phylum Firmicutes and the class *Mollicutes*. The Mycoplasma genus has genomes ranging from 580 to 2,200 kpb in size, with that of *M. hyopneumoniae* measuring 900 kpb and 300 to 900 nm in diameter. They lack a cell wall, have varied morphology, and are enveloped by a 10 nm thick plasma membrane. Their molecular biology is similar to that of gram-positive bacteria (Kobisch and Friis, 1996; Simionatto et al., 2013). *M. hyopneumoniae* is a globally distributed pathogen specific to domestic pigs (*Sus scrofa domesticus*) and wild boars (*Sus scrofa scrofa*) (Maes et al., 2008). EP is a chronic (Pieters et al., 2009), slowly spreading infectious disease (Roos et al., 2016) characterized by bronchopneumonia. Clinically, it manifests as a dry cough, delayed weight gain, high morbidity, and low mortality (Kuhnert and Overesch, 2014; Maes et al., 2017).

The factors contributing to the severity of EP include management and housing conditions, as well as the virulence of the infecting strain. Variations in virulence have been demonstrated among different field isolates of *M. hyopneumoniae*, leading to a classification of low, medium, and high pathogenicity based on clinical signs and lesions (Vicca et al., 2003; Meyns et al., 2007; dos Santos et al., 2015; Leal Zimmer et al., 2020). *M. hyopneumoniae* employs various mechanisms of pathogenicity such as cell adhesion (Siqueira et al., 2014; Raymond and Djordjevic, 2015; Leal Zimmer et al., 2020), secretion, signaling (Beuckelaere et al., 2022), cytotoxicity, apoptosis, and immunomodulation (Bai et al., 2013; Ni et al., 2015; Deeney et al., 2019; Yu et al., 2020), some of which are not fully understood (Leal Zimmer et al., 2020). Due to the uniqueness of mycoplasmas compared to other studied pathogens, there is limited opportunity to identify known homologous genes in other pathogens (Rycroft, 2020).

*Mycoplasma hyopneumoniae* adheres to respiratory epithelial cells, induces infiltration of macrophages and lymphocytes, and triggers the accumulation of inflammatory cells, leading to an inflammatory response (Woolley et al., 2012). This process destroys the mucociliary apparatus of the respiratory epithelium, alters the cell architecture, and results in ciliary loss, predisposing individuals to secondary infection (Woolley et al., 2012; Bustamante-Marin and Ostrowski, 2017). Several researchers described and studied mechanisms contribute to *M. hyopneumoniae* ability to colonize the respiratory tract and actively excrete the pathogen over extended periods (Leal Zimmer et al., 2020).

Colonization of the respiratory ciliated epithelium by *M. hyopneumoniae* specifically depends on the expression of two functionally redundant adhesin families, P97 and P102 (Siqueira et al., 2014). Approximately 20–30% of the genes in *M. hyopneumoniae* are responsible for surface protein expression, although many of their functions remain unknown (Felde et al., 2018). Adhesins are crucial, with at least 35 proteins are associated with cell adhesion, including several related to the P97/P102 families (Zhang et al., 1995; Bogema et al., 2012; Siqueira et al., 2014; Raymond and Djordjevic, 2015; Leal Zimmer et al., 2020).

Up to the present moment, 26 genomes deposited at NCBI can be found completely sequenced. The *M. hyopneumoniae* strain J was obtained by Friis in the United Kingdom in 1957 as first isolated (Kobisch and Friis, 1996) and its genome was sequenced in 2005 (Vasconcelos et al., 2005). The other isolates sequenced originate 232 United States (Minion et al., 2004); 7,422 and 7,448 Brazil (Vasconcelos et al., 2005; Siqueira et al., 2013); 168/168-L China (Liu et al., 2011); 11 Netherlands (Kamminga et al., 2017); 98 Netherlands; F7.2C Switzerland (Trueeb et al., 2019); KM014 South Korea (Han et al., 2017); LE China (Xie et al., 2021); TB1 China (Qiu et al., 2019); ES2-L China (Zong et al., 2022) ES2 China; MHP699, MHP650, MHP653, MHP679, MHP682, MHP691, MHP694, MHP709, MHP696 France; NCTC10127 UK and UFV01/UFV02 Brazil.

In Brazil, few studies have reported on the characterization of *M. hyopneumoniae* strains in the country (Yamaguti, 2009; dos Santos et al., 2015; Andrade et al., 2023). Currently, only isolates 7,422 and 7,448 have been completely sequenced and deposited in the NCBI by another research group (Vasconcelos et al., 2005; Siqueira et al., 2013).

Therefore, the objective of this study is to perform a comparative genomic analysis between the UFV01 and UFV02 isolates, as well as with others deposited in the NCBI database. Additionally, the aim is to induce infection in pigs using these two specific *M. hyopneumoniae* isolates to characterize virulence through clinical signs and induced lesions, and to identify variations in antibody levels, DNA load and cytokines.

## 2 Materials and methods

### 2.1 Origin and cultivation of the UFV01 and UFV02 isolates

The isolates were obtained through the collection of lungs suspected of EP from slaughtered animals originated from a property located in the Piranga Valley in the state of Minas Gerais, Brazil (Gonzaga et al., 2019). The isolation for obtaining pure cultures was performed following the standardized methodology by Cook et al. (2016).

The isolates were cultured in 1x Friis medium (liquid) and 2.8x Friis medium (solid) and prepared according to Cook et al. (2016). They were reactivated in a liquid medium at a ratio of 1:9 (frozen inoculum to medium). The test tubes were incubated at 37°C and observed daily until a color change occurred. Bacterial growth was evidenced by the alteration of the medium’s color. Three passages were performed to reactivate the isolate, and after the fourth passage, the inoculum was aliquoted into individual volumes of 10 mL and frozen at −80°C until the time of inoculation. The isolates were quantified using the Color Changing Units (CCU) technique, performed according to Calus et al. (2010). In parallel, the isolates were cultured on 2.8x solid Friis medium to visualize the colony morphology. The isolates were incubated for seven days in an oven at 37°C as described by Cook et al. (2016).

### 2.2 Whole genome sequence analysis of UFV01 and UFV02

The DNA extraction of the isolates (UFV01 and UFV02) was conducted using a Wizard® Genomic DNA Purification Kit (PROMEGA^®^, United States) following the manufacturer’s recommendations. A private company (MicrobesNG/United Kingdom) performed the quantification, library preparation, and sequence steps. The raw data (pair-end 150 bp) were trimmed using the Trimmometics software (Bolger et al., 2014) and quality was analyzed using FastQC (Andrews, 2017). The contigs were assembled by the *De novo* technique, using the SPADES 3.15.3, MIRA 4.9.6, and A5-Miseq software (Chevreux et al., 2004; Tritt et al., 2012; Nurk et al., 2013), and the results were evaluated by with the Quality Assessment Tool for Genome Assemblies – Quast (Gurevich et al., 2013) to choose the best assembly.

The annotation and subsystem classification were made using Prokka 1.14.6 (Seemann, 2014), the Bacterial and Viral Bioinformatics Resource Center (BV-BRC) platform (Olson et al., 2023) and the files built by the Genbank deposit. The virulence factors, toxin gene prediction, resistance genes, and sequence typing (ST) were analyzed using the online platforms Virulence Factors of Pathogenic Bacteria – VFPB,^1^ PathoFact version 1.0 (de Nies et al., 2021), the Comprehensive Antibiotic Resistance Database – CARD tools (McArthur et al., 2013), and pubMLST (Jolley et al., 2018) for sequence type determination, considering a cutoff of 70% for the scores of identity and similarity to determine their presence in the genetic system of the bacteria. The genome comparison was performed using BLAST Ring Image Generator (BRIG) (Alikhan et al., 2011) and Anvio-7.1 (Delmont and Eren, 2018; Eren et al., 2020). Orthologue group genes were analyzed and compared using the Orthofinder 2.5.2 software (Emms and Kelly, 2019). The two isolates were compared with 22 assemblies from the NCBI database. The strains used were 98 (Access Number: ASM1341272v1), ES-2 (Access Number: ASM476872v1), ES-2 L (Access Number: ASM1340275v1), F7.2C (Access Number: ASM792398v1), J (Access Number: ASM820v1), KM014 (Access Number: ASM225750v1), MHP650 (Access Number: ASM983217v1), MHP653 (Access Number: ASM983194v1), MHP679 (Access Number: ASM983212v1), MHP691 (Access Number: ASM983208v1), MHP694 (Access Number: ASM983190v1), MHP696 (Access Number: ASM983203v1), MHP699 (Access Number: ASM983185v1), MHP709 (Access Number: ASM983207v1), NCTC10127 (Access Number: 51334_A01-3), LH (Access Number: ASM2138386v1), 168 (Access Number: ASM18318v1), 168 L (Access Number: ASM40085v1), 232 (Access Number: ASM840v1), 7,422 (Access Number: ASM42721v1), 7,448 (Access Number: ASM822v1), and TB1 (Access Number: ASM221348v1) (Supplementary material S1).

### 2.3 Experimental design and sample collection

Approximately 23-day-old Landrace x Large White piglets, with an average weight of 7.585 ± 0.591 kg, were acquired from the Agroceres PIC company; all tested negative for *M. hyopneumoniae*. All procedures were performed following the approval of the code of conduct for the use of animals in teaching, research, and extension of the Federal University of Viçosa, regulation 39/2021.

After the arrival of the animals, they underwent a 12-day adaptation period in the facilities. All animals tested negative in the qPCR (Fourour et al., 2018) for agent detection and for the presence of anti- *M. hyopneumoniae* (IgG) antibodies in ELISA *M. hyopneumoniae* Ab test (IDEXX, United States). The study was divided into two test groups (*n* = 8 each) and one negative control group (*n* = 4), totaling 20 animals. One test group was inoculated with the UFV01 isolate, the second group was inoculated with the UFV02 isolate, and the negative control group was inoculated with the Friis medium. The animals were kept in disinfected pens and isolated from the external environment in the isolation unit.

After the 12-day acclimation period, at 35 days of age, the animals in the test groups were intratracheally challenged with 10 mL of inoculum containing 1×10^6^ CCU/mL. The negative control group was inoculated with 10 mL of Friis medium. Throughout the entire experimental period, no form of antimicrobial agent was administered through either water or food.

On day 0 (day inoculation), and 7, 14, 21, 28, and 35 days post inoculation, serum and laryngeal swab samples were collected to assess the seroconversion curve and agent excretion. On 35 dpi, all animals were euthanized. Bronchoalveolar lavage fluid (BALF) samples were collected at 35 dpi, immediately after euthanasia.

Duplicate samples of lung lesions for qPCR analysis were collected from portions with suggestive lesions of porcine EP and stored in cryotubes, then kept in liquid nitrogen (−196°C) until DNA extraction.

### 2.4 Evaluation of the clinical signs

Following the challenge, a daily monitoring routine was implemented in the morning for a duration of 40 min. This monitoring involved observing the animals for 20 min while they were agitated in the stall. Then, the animals were evaluated for 20 min under rest conditions. Incidences of non-productive dry cough episodes occurring during this specified period were systematically quantified and documented by a single observer, who remained blinded to the study’s conditions, in accordance with the research conducted by Silva et al. (2022).

### 2.5 Cytokine evaluation

The tests for cytokine quantification in serum were performed using the Invitrogen IL-10 Porcine ELISA Kit, IFN-gamma Porcine ELISA Kit, and TNF-alpha Porcine ELISA Kit (Thermo Fisher Scientific^®^, Wilmington, DE, United States). These kits are based on a standard curve within a range of 500 to 7.8 pg./mL for IL-10 and IFN-gamma, and 1,500 to 23.4 pg./mL for TNF-alpha. The samples were tested on two plates. The standard curve was determined by the ELISA reader program and constructed in 4-parameter mode, with the concentration determined kit provided values and according to the quantification obtained in the tests.

### 2.6 ELISA for detection of anti- *Mycoplasma hyopneumoniae* IgG and IgA

Blood collection was performed on 0, 7, 14, 21, 28, and 35 dpi from the orbital sinus. The collected samples were centrifuged at 8,000 g for 15 min, and 1 mL of serum was stored and kept at −20°C until use. Anti- *M. hyopneumoniae* IgG antibodies were detected from serum samples using the *M. hyopneumoniae* Ab test (IDEXX, United States), which is a commercial indirect ELISA kit (99.6% specificity and 89.4% sensitivity). The test was conducted following the manufacturer’s recommendations. The S/P calculation was also performed according to the manufacturer’s instructions. As this was an experimental infection, S/*p* values above 0.3 were considered positive. For the IgA ELISA in BALF, we used the methodology standardized by Mechler-Dreibi et al. (2021). The test is based on the use of reagents and plates from the commercial *M. hyopneumoniae* Ab kit (IDEXX, United States), with some modifications.

### 2.7 Detection of *Mycoplasma hyopneumoniae* DNA by real time qPCR

The laryngeal swab, lung tissue, and BALF samples had their DNA extracted using the adapted methodology described by Kuramae-Izioka (1997). As endogenous controls for the DNA extractions from the samples, a primer designed by Assao et al. (2019) was used, which amplifies a 107 bp region of the 18S ribosomal gene (GenBank: AY265350.1).

For the detection of the *M. hyopneumoniae* genome in the samples, the set of primers and probes described by Fourour et al. (2018) was used: forward primer: 5’ TAAGGGTCAAAGTCAAAGTC-3′, reverse primer: 5′- AAATTAAAAGCTGTTCAAATGC-3′, and hydrolysis probe: 5′- FAM-AACCAGTTTCCACTTCATCGCC-BHQ2-3′. A fragment of *M. hyopneumoniae* DNA was amplified by conventional PCR and cloned using the CloneJET PCR Cloning Kit (Thermo Fisher^®^) for the construction of the standard curve. All samples were tested in duplicate, and those showing a variation greater than 0.5 Ct were tested again in triplicate.

The reaction consisted of 10 μL of 2X iTaqTM Universal Probes Master Mix (Bio-Rad, California, EUA), 1 μL of each primer at 10 nmol/μl (Invitrogen, USA), 0.6 μL of the hydrolysis probe at 10 nmol/μl (IDT, Iowa City, USA), 5.4 μL of ultrapure water, and 2 μL of DNA per sample, totaling 20 μL of reaction. The qPCR was performed on a CFX-96 real-time thermocycler (Biorad, United States) under the following conditions: an initial denaturation cycle at 95°C for 3 min, followed by 39 cycles of 95°C for 15 s and annealing/extension at 55.7°C for 1 min (Almeida et al., 2020).

### 2.8 Macroscopic and microscopic lesions

After the euthanasia of the animals at 35 days of infection, lung lesions compatible with swine EP were scored according to the methodology described by Straw et al. (1986). Each of the lung lobes was assessed macroscopically and the percentage of surface area affected by lesions was estimated. The value was multiplied by the weight that each lung lobe represented in relation to the total surface area the lesions can range from 0% (no lesions) to 100% (entire lung affected). The evaluated characteristics included red and firm consolidation in the apical, cardiac, accessory, and diaphragmatic lobes.

Histological slides were prepared for semiquantitative classification of microscopic lesions. The method to mensurate the lesions was adapted from a study published by Hansen et al. (2010). The sections were systematically examined by evaluating the following structures in each section: bronchi, bronchioles, bronchus-associated lymphoid tissue (BALT), alveolar ducts, and alveoli, including alveolar septa, peribronchial, peribronchiolar, and interlobular connective tissues, and pleura. BALT hyperplasia was graded as follows: (−) absent; (1) mild, diffuse infiltration of lymphocytes in peribronchial, peribronchiolar, and perivascular tissues, including the lamina propria of the airways; (2) moderate increase in diffuse lymphocyte infiltration and/or presence of some lymphoid nodules; and (3) with marked the number of lymphoid nodules. Bronchopneumonia and pleuritis lesions were graded in the same way, according to the intensity of the lesions. The pathologists were blinded to assess clinical and lesions data.

### 2.9 Differential diagnosis

Individual lung swabs were collected from the animals and subjected to total RNA extraction using the commercial RNA extraction kit from Promega United States – ReliaPrep^™^ RNA Miniprep Systems for the diagnosis of the Influenza A virus using conventional PCR methodology. The primers used in the PCR reactions as a reference were described by Fouchier et al. (2000). For the detection of the bacterial agents (*Glaesserella parasuis*, *Pasteurella multocida*, *Mycoplasma hyorhinis*, *Streptococcus suis*, and *Actinobacillus pleuropneumoniae*), the qPCR technique was used with DNA samples obtained from the BALF, following the methodologies previously described (Bonifait et al., 2014; Fourour et al., 2018; Goecke et al., 2020; Han et al., 2020; Sunaga et al., 2020), respectively (Supplementary material S2).

### 2.10 Statistical analysis

The quantitative variables were assessed using the Shapiro–Wilk test to check for the normality of errors and the Levene test to assess the homogeneity of variances. Data that did not meet the assumptions of normality and homoscedasticity were subjected to statistical transformations. The degrees of freedom for repeated measures ANOVA were adjusted using the Greenhouse–Geisser correction for the factors of day and the day x treatment interaction. Non-parametric data were subjected to the Wilconxon test with significance of *p* < 0.001.

The data were evaluated using linear models with repeated measures over time, and pairwise comparisons between means were performed using the Tukey’s test (*p* < 0.05). Statistical analyzes were conducted using R software, version 4.2.1 (R Core Team, 2022).

## 3 Results

### 3.1 WGS analysis

**Figure 1:**
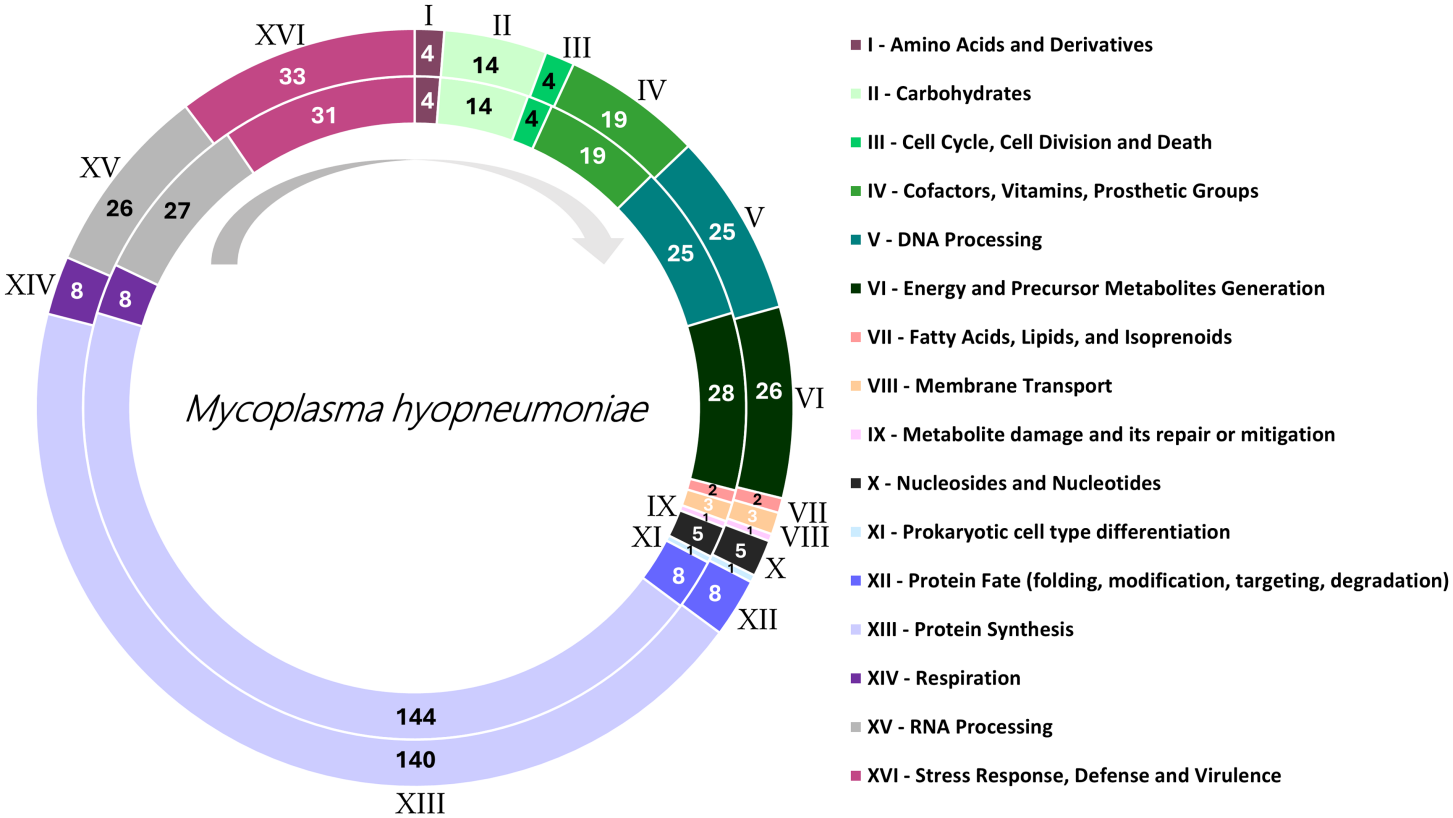
Sixteen class category distributions of UFV01 (internal circle) and UFV02 (external circle), annotated by Bacterial and Viral Bioinformatics Resource Center (BV-BRC) using as reference the taxonomy ID: 2099 – *Mesomycoplasma hyopneumoniae*. Each color and Roman numeral identify different function Class, while the numbering inside the circles represents number of genes. The arrow in gray suggests how the caption should be read.

The genomes UFV01 (NCBI: PRJNA542605), and UFV02 (NCBI: PRJNA542605) were previously deposited in the NCBI, with UFV01 presenting 909.816 pair bases, 28.4% G + C content, and 751 CDS; UFV02 presented 959.419 pair bases, 28.5% GC content and 788 CDS. Features such as genome size, number coding system, and GC content were like those of other genomes in the NCBI library. For the CDS and subsystem identification, the protein files obtained from GenBank were used in the BV-BRC platform annotation showed differences in the number of coding sequences and subsystem features (Figure 1), accounting for 58–60% for the CDS identified and 16 predicted subsystems important to classify the genes according to their genes.

UFV01 and UFV02 presented 16 different function classes (Figure 1). Inside of the classes, UFV02 presented 324 annotated genes, and 81 subsystems. In contrast, UFV01 presented 319 annotated genes, thus denoting more genes attributed to the former. Moreover, UFV01 had fewer annotated genes related to protein synthesis (Figure 1-XIII), with four less than UFV02. In addition, it presented two genes less for energy and precursor metabolites generation (Figure 1-VI) and one gene less for RNA Processing (Figure 1-XV). However, it showed two more genes related to Stress Response, Defense, and Virulence (Figure 1-XVI). The smaller number of annotated genes could be related to the larger number of genes with still unidentified characteristics.

The virulence factors identified for both isolates were *mhp385, mhp271, MHP_RS03455, p102, p97, p216, MHP_RS00555,* and *mhp107,* and the characterized toxins were *tlyC* (putative hemolysin), *PLDc_2* (PLD-like domain) and *hcnC* (hydrogen cyanide synthase HcnC). The sequence type for these two isolates characterized using the PubMLST platform was the same and was classified as ST-123. No resistance genes were found in these isolates, except for the isolate UFV02, which presented the gene *KpnF* related to efflux pumps for different compounds (macrolide, aminoglycoside, cephalosporin, tetracycline, peptide antibiotic, rifamycin, disinfecting agents and antiseptics).

**Figure 2:**
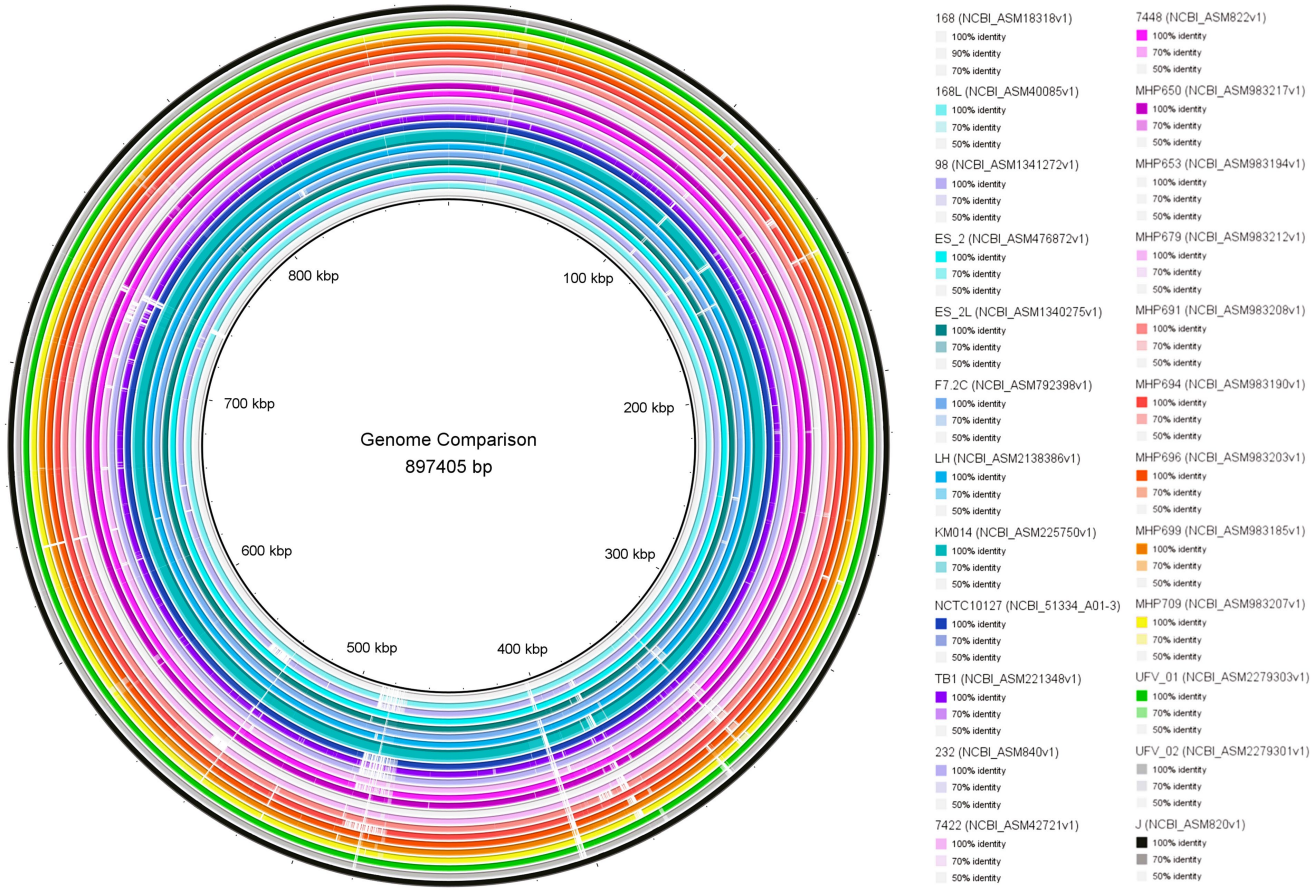
Comparison of *Mycoplasma hyopneumoniae* isolates UFV01 and UFV02 with 22 references assemblies from NCBI using the BLAST Ring Image Generator (BRIG) (Alikhan et al., 2011).

**Figure 3:**
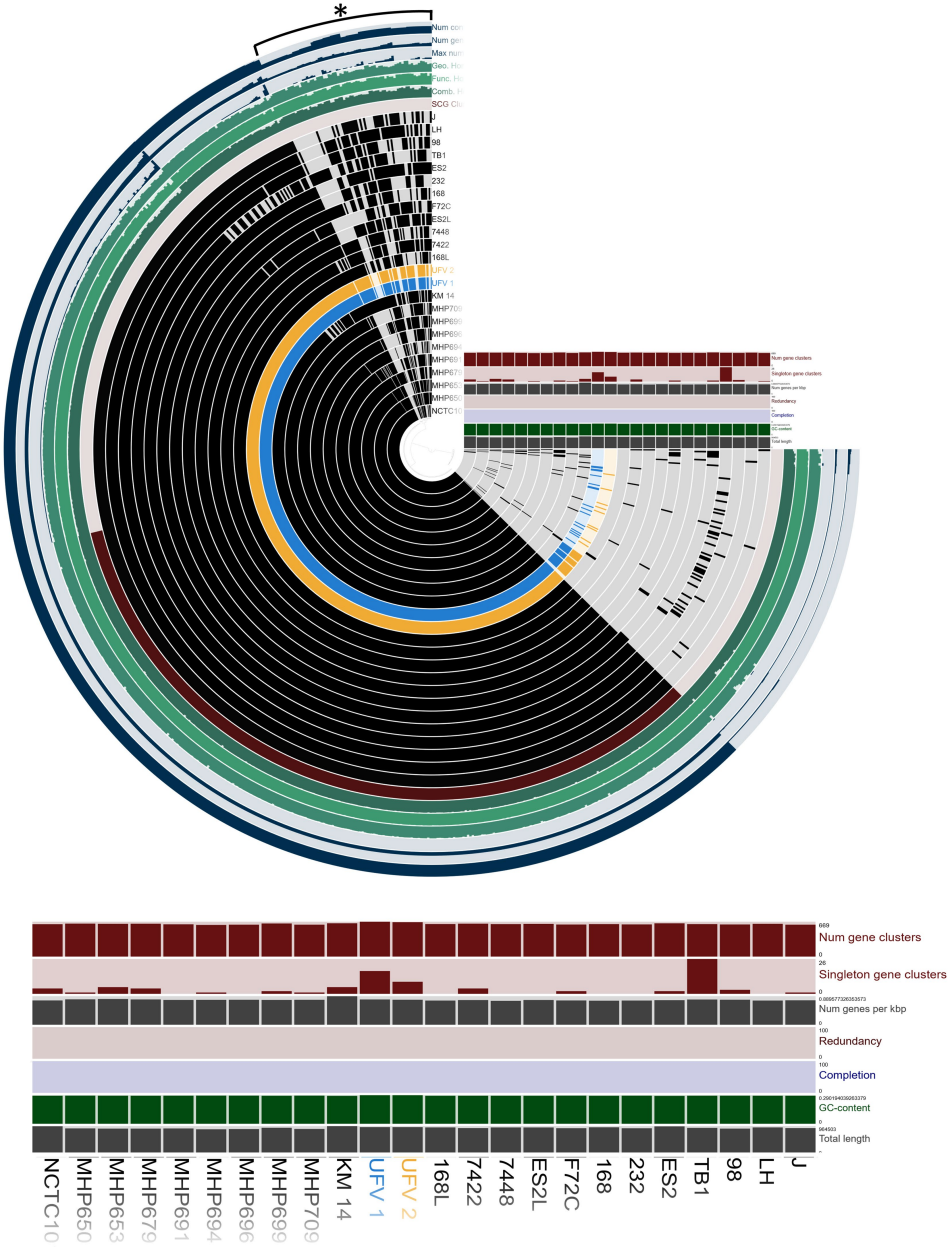
Pangenome of 22 reference assemblies downloaded from NCBI and the two isolates, UFV01 (blue) and UFV02 (yellow), using Anvio-7.1 (Delmont and Eren, 2018; Eren et al., 2020). The figure illustrates the core-genome comparison, G + C content, length of genome, number of gene clusters, and singleton gene cluster. *Variable gene cluster.

The genome comparison is shown in Figure 2. Overall, UFV01 and UFV02 present genomes with few gaps compared to other genomes. Some gaps seem to be present in all genomes when compared to the J strain (analysis reference). Comparing the core-genome analysis (Figure 3), UFV01 presents more exclusive genetic clusters (singletons) that are individual genes without any homologs compared to UFV02. However, the genome of TB1, which is of Chinese origin, had the most compared to all other genomes in this study.

**Figure 4:**
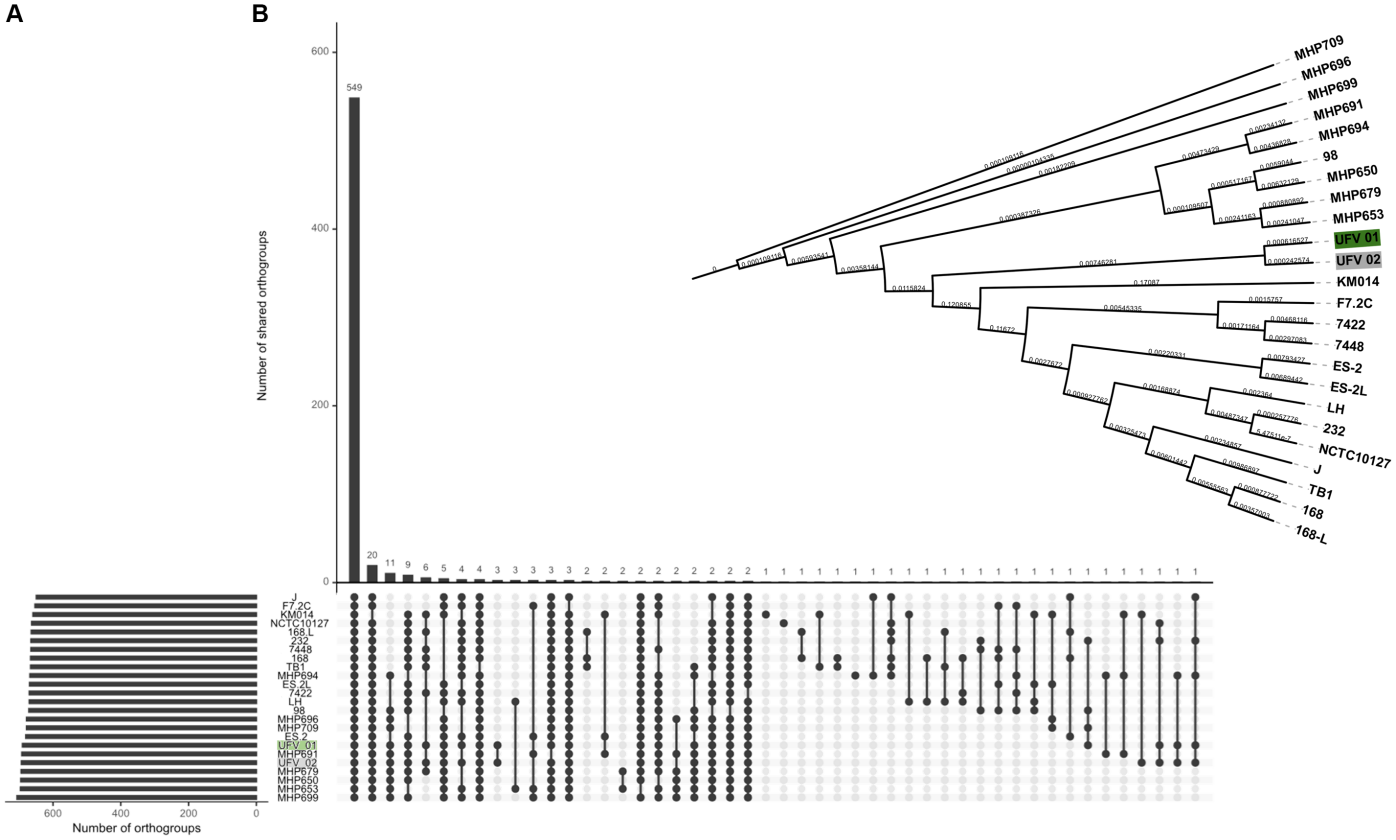
Orthogroup comparison of 22 NCBI reference assemblies and the isolates UFV01 (green) and UFV02 (gray), analyzed by the Orthofinder 2.5.2 software (Emms and Kelly, 2019). **(A)** The common and uncommon orthogroups from all genomes **(B)**. Dendogram based on the presence or absence of orthogroups.

The orthogroup analysis (Figure 4) showed that all genomes share 549 groups. For UFV01 and UFV02 specifically, a total of 03 orthogroups was reported only in these two isolates, and these strains seem to have few more orthogroups compared to the other genomes (Figure 4A). Considering all genomes in this genetics group, the UFV01 and UFV02 seems in the middle of the tree, being um side close of MPH groups strains (French), and the other side close of KM014 (South Korea), F7.2C (Belgium) and 7422/7448 (Brazil) strains (Figure 4B).

### 3.2 Evaluation of the clinical signs and lung lesions

**Figure 5:**
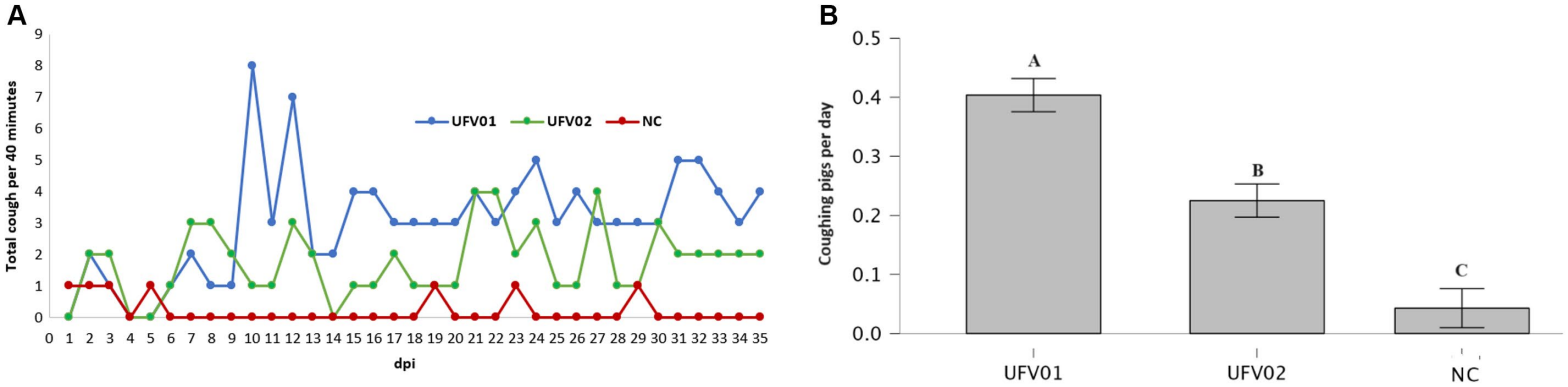
**(A)** Cough frequency assessment graph. Orange line – Control group. Green line – UFV01 group. Blue line – UFV02 group. NC – negative control; dpi – days post inoculation. **(B)** Average daily number of coughs per animal in each experimental group over the 35-day challenge period, measured during 40-min day. Different letters indicate statistical significance based on the Wilcoxon test with a significance level of *p* < 0.001.

The cough frequencies of the control, UFV01, and UFV02 groups were observed daily, as described in the methodology. From the first week onwards, animals in the UFV01 and UFV02 groups started to exhibit coughing episodes consistent with EP. The UFV01 group exhibited a higher numerical count of recorded coughs per 40 min in comparison to the UFV02 group at certain intervals (Figure 5A). When evaluating the release media for a duration of 40 min daily, considering the number of animals per group over the entire experimental period, all groups displayed statistically significant differences from one another. Specifically, the UFV01 group showed 0.44 coughs per pig per 40 min, while the UFV02 group exhibited 0.22, and the NC group registered 0.04 (Figure 5B).

**Figure 6:**
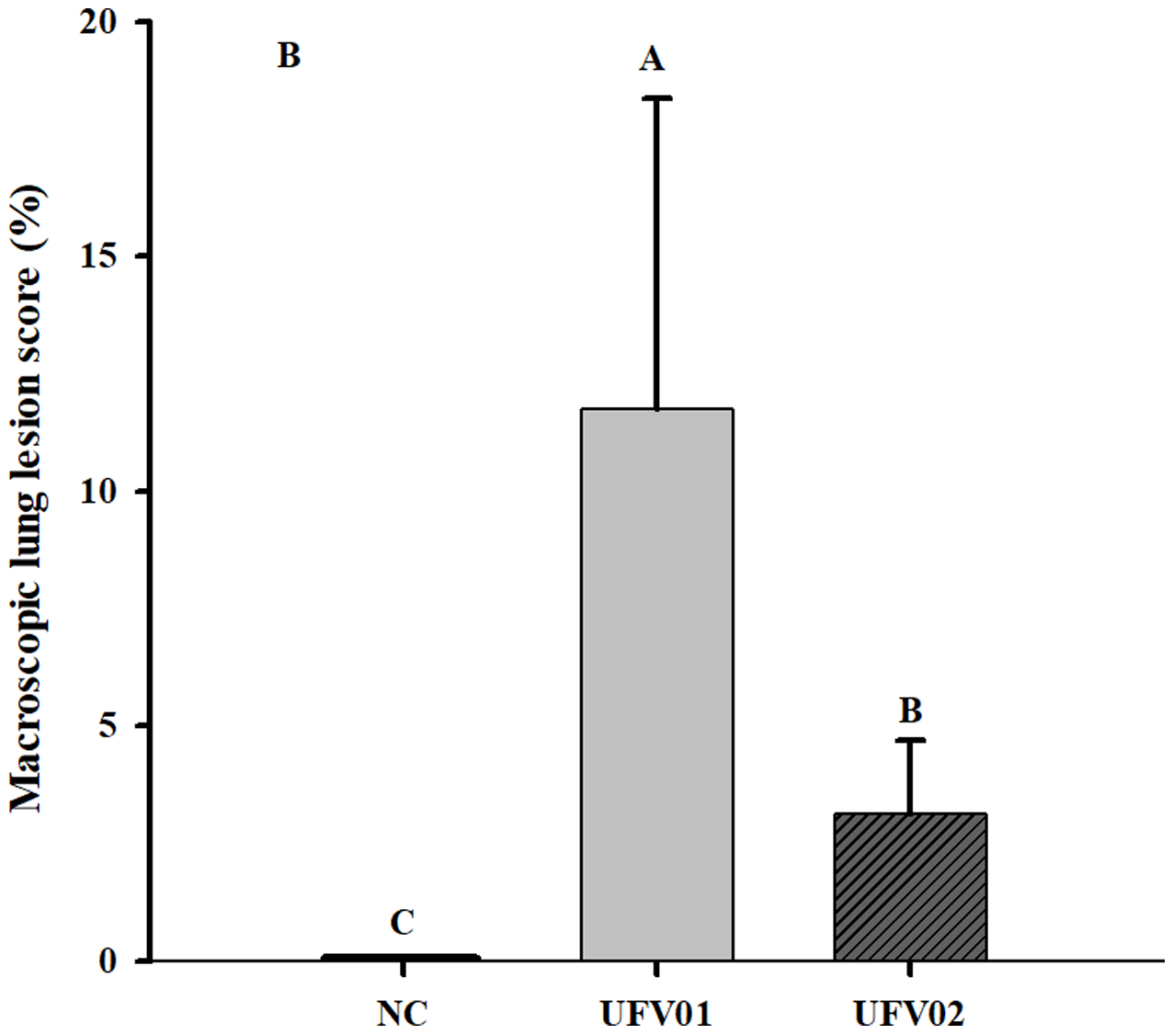
Bar graph on a scale of 0% (no lesions) to 100% (entire lung affected). *Different letters indicate significant differences detected in the Tukey’s test (*p* < 0.05).

**Figure 7:**
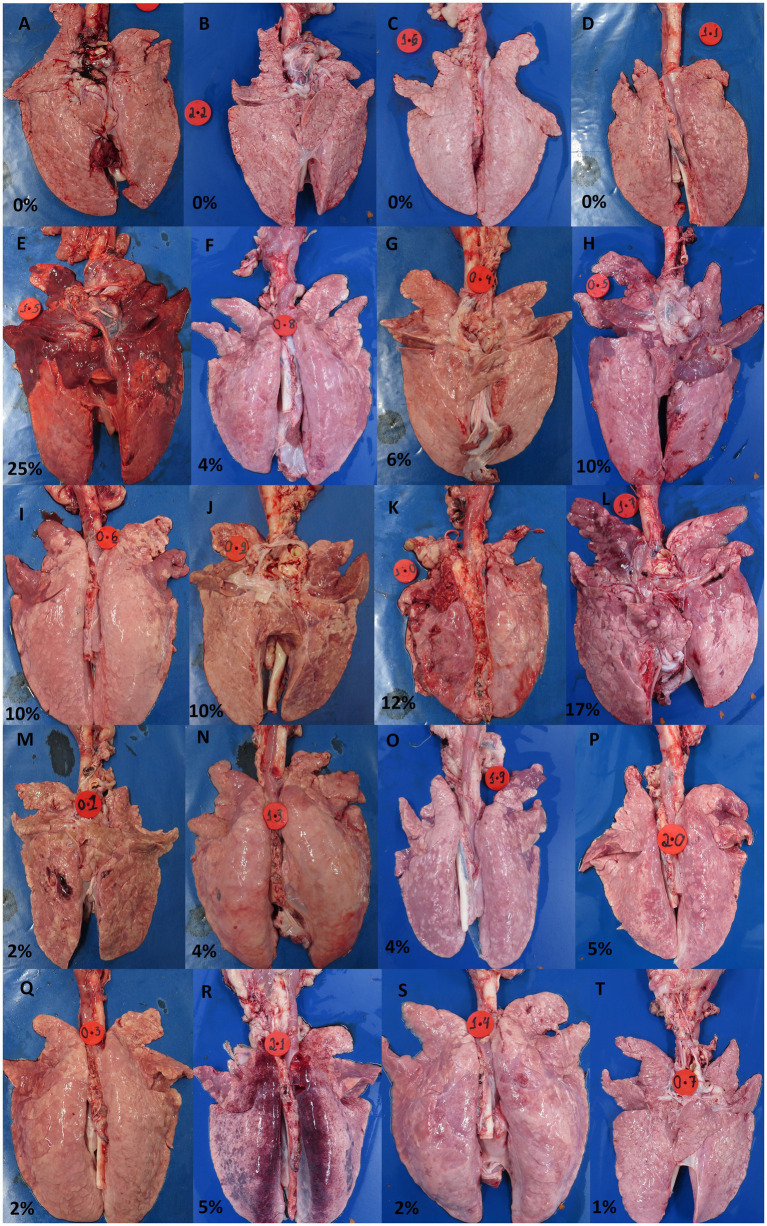
Individual photos of the lungs from the control group at 35 dpi **(A–D)**, lungs from the group inoculated with the UFV01 strain at 35 dpi **(E–L)**, and lungs from the UFV02 group at 35 dpi **(M–T)**.

Macroscopic lesions characteristic of EP in the lungs were scored from 0 to 100% (indicating the extent of tissue affected). As can be observed in Figure 6, the group inoculated with the UFV01 strain exhibited more severe macroscopic lesions (*p* < 0.05). Specifically, the UFV01 group (Figures 7E–L) presented 11.75% (±6.60) macroscopic lesions at 35 dpi (Figure 6), and the UFV02 group (Figures 7M–T) exhibited 3.125% (±1.55) lesions. The animals in the control group (Figures 7A–D) showed 0% macroscopic lesions.

The microscopic lesions found in the processed fragments consisted of bronchopneumonia, BALT hyperplasia, and pleuritis. They were scored according to Table 1 as absent (−), mild (1), moderate (2), and severe (3). Table 1 presents the frequencies of each respective lesion and its degree of involvement. Animals in the control group (NC) had no lesions. Animals in the UFV01 group had 50% mild bronchopneumonia lesions (4/8), 37.5% moderate lesions (3/8), and 12.5% severe lesions (1/8). Regarding the BALT analysis, 50% had moderate lesions (4/8) and 50% had severe lesions (4/8). In the UFV02 group 62.5% of the pigs had mild bronchopneumonia lesions (5/8) and 37.5% moderate lesions (3/8). The BALT analysis 62.5% mild lesions (5/8) and 25% moderate lesions (2/8) and one animal (1/8) in the UFV02 group showed no lesions. Only the UFV01 group had cases of pleuritis, with 25% mild (2/8) and 12.5% moderate (1/8).

**Table 1:** Description of quantified microscopic lesions (bronchopneumonia, BALT hyperplasia, and pleuritis).

Grupo	Animal	Bronchopneumonia	BALT Hyperplasia	Pleuritis
NC	A	–	–	–
	B	–	–	–
	C	–	–	–
	D	–	–	–
Score average		0.00	0.00	0.00
UFV01	E	3	1	2
	F	1	1	-
	G	1	2	-
	H	1	2	1
	I	2	2	-
	J	2	1	-
	K	2	2	-
	L	1	1	1
Score average		1.62	1.5	0.5
UFV02	M	2	1	–
	N	1	2	–
	O	2	1	–
	P	2	1	–
	Q	1	1	–
	R	1	2	–
	S	1	1	–
	T	1	–	–
Score average		1.37	1.12	0.00

Absence of lesions (-), mild lesion (1), moderate lesion (2), and severe lesion (3).

When comparing the mean scores of the lesions according to the scoring described in Table 1, the UFV01 group scored 1.62 for bronchopneumonia, 1.5 for BALT hyperplasia and 0.5 for pleuritis. On the other hand, the UFV02 group scored 1.37, 1.12, and 0.0, respectively, for the three aforementioned lesions (Table 1).

### 3.3 ELISA IgG and IgA

**Figure 8:**
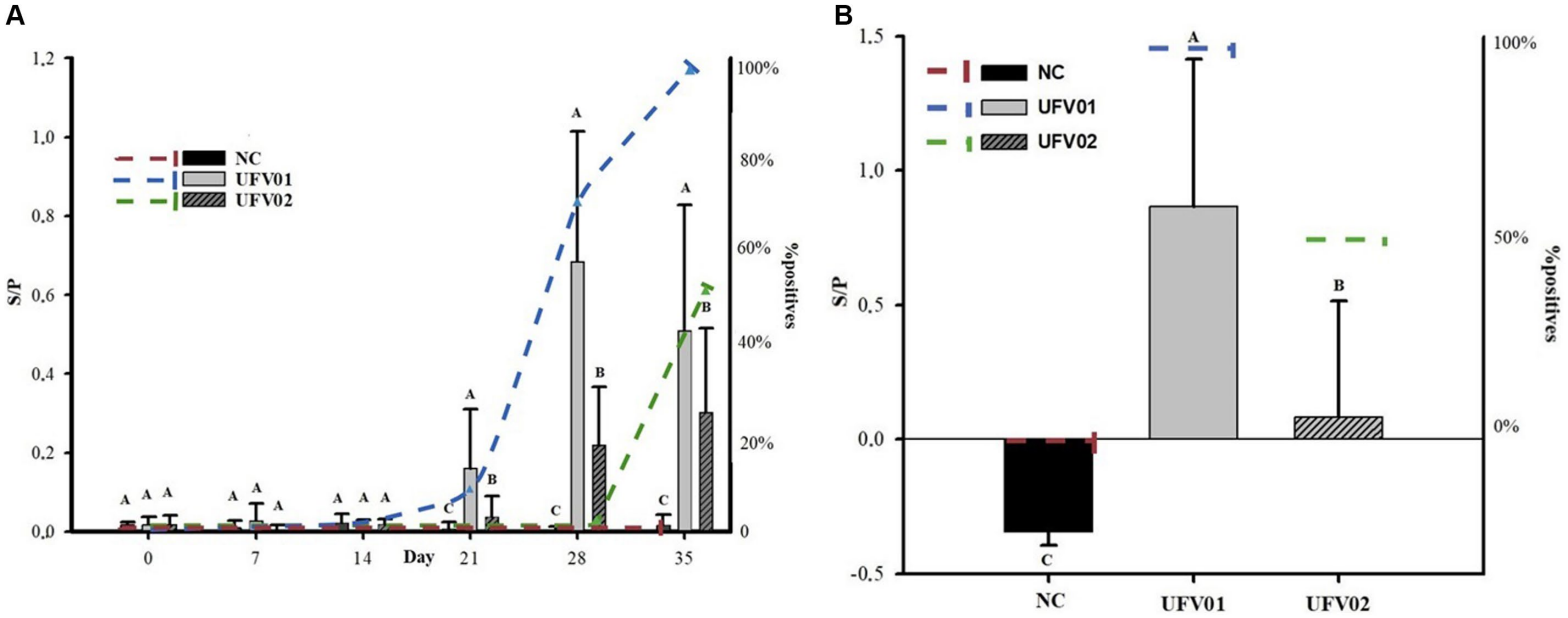
**(A)** Dynamics of IgG antibody detection and the number of positive animals during the experimental course of pigs inoculated with *Mycoplasma hyopneumoniae* using UFV01 and UFV02 isolates. **(B)** Dynamics of IgA antibody detection in BALF and the number of positive animals at 35 days after experimental inoculation with *Mycoplasma hyopneumoniae* using UFV01 and UFV02 isolates. Different letters indicate significant differences detected in the Tukey’s test (*p* < 0.05). *The bars indicate antibody levels (S/P) and the dashed lines indicate the percentage (%) of positive animals.

The animals in the control group remained negative throughout the study. The detection of IgG dynamics of both groups was not uniform. In the UFV01 group, seroconversion began at 21 dpi with 12.5% (1/8) of the animals, increased to 75% (6/8) at 28 dpi and reached 100% (8/8) by 35 dpi. In contrast, in the UFV02 group, only 50% (4/8) had seroconverted at 35 dpi (Figure 8A). The antibody levels generated by UFV01 were statistically higher than those of UFV02 at 21, 28, and 35 dpi, as observed in Figure 8A.

An analysis was conducted to detect IgA antibodies in the BALF of the three groups. No anti- *M. hyopneumoniae* IgA were detected in the NC group. In the UFV01 group, 100% (8/8) of the individuals tested positive, while only 50% (4/8) tested positive in the UFV02 group, as observed in Figure 8B. The animals that tested positive for IgA in the UFV02 group were the same individuals that tested positive for IgG.

### 3.4 qPCR of BALF, laryngeal swabs and lung tissue

**Figure 9:**
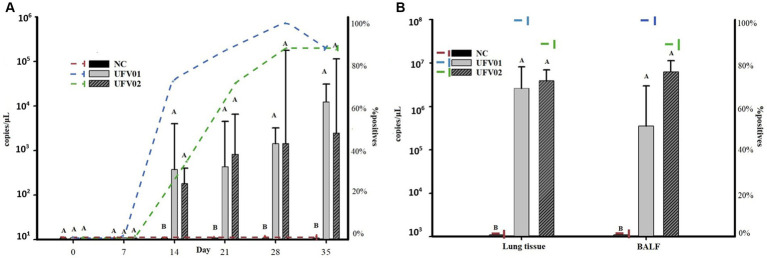
**(A)** Dynamics of the detection of *Mycoplasma hyopneumoniae* P102 gene fragment copies in laryngeal swabs and the number of pigs testing positive after experimental inoculation with the UFV01 and UFV02 isolates. **(B)** Dynamics of the detection of copies of the P102 gene fragment of *Mycoplasma hyopneumoniae* in BALF and lung tissue, and the number of experimentally inoculated pigs testing positive for the UFV01 and UFV02 isolates. Different letters indicate significant differences detected in Tukey’s test (*p* < 0.05) for the number of *M. hyopneumoniae* copies. *Bars indicate the number of copies per microliter of DNA detected, and the dashed lines show the percentage (%) of positive animals.

Laryngeal swabs were collected and subjected to qPCR analysis to evaluate the positivity and bacterial load excreted by the groups. The onset of bacterial shedding occurred at 14 dpi. As observed in Figure 9A and Supplementary material S3, the UFV01 group had a positivity of 75% at 14 dpi (3.76×10^2^ copies/μL), 87.5% at 21 dpi (4.36×10^2^ copies/μL), 100% at 28 dpi (1.44×10^3^ copies/μL), and 87.5% at 35 dpi (1.25×10^4^ copies/μL). The UFV02 group also started shedding bacteria at 14 dpi, but with a positivity of 37.5% (1.82×10^2^ copies/μL), 75% at 21 dpi (8.33×10^2^ copies/μL), 87.5% at 28 dpi (1.44×10^3^), and 87.5% at 35 dpi (2.50×10^3^ copies/μL). It should be noted that one individual in the UFV02 group (T) tested negative on all days.

Regarding the bacterial loads shed throughout the experimental course, it can be observed in Figure 9A that both challenged groups (UFV01 and UFV02) differed statistically from the negative control group only from 14 to 35 dpi. Both test groups showed a numerical increase in DNA copy excretion from 14 to 35 dpi (Figure 9A; Supplementary material S3), but did not differ statistically from each other.

BALF and lung tissue samples were subjected to qPCR analysis to evaluate the bacterial load present in the respiratory system of the animals at 35 dpi, during euthanasia. As shown in Figure 9B, the NC group tested negative in both analyzes, confirming once again their complete negativity. The UFV01 group showed 100% positivity (8/8) in both the BALF and tissue analyzes, with respective average bacterial loads of 3.57×10^5^ copies/μL and 2.63×10^6^ copies/μL of DNA. The UFV02 group had 87.5% positivity in both assessments, with an average bacterial load of 6.32×10^6^ copies/μL and 3.92×10^6^ copies/μL of DNA, respectively. See Supplementary material S3 for more details.

### 3.5 Cytokines

**Figure 10:**
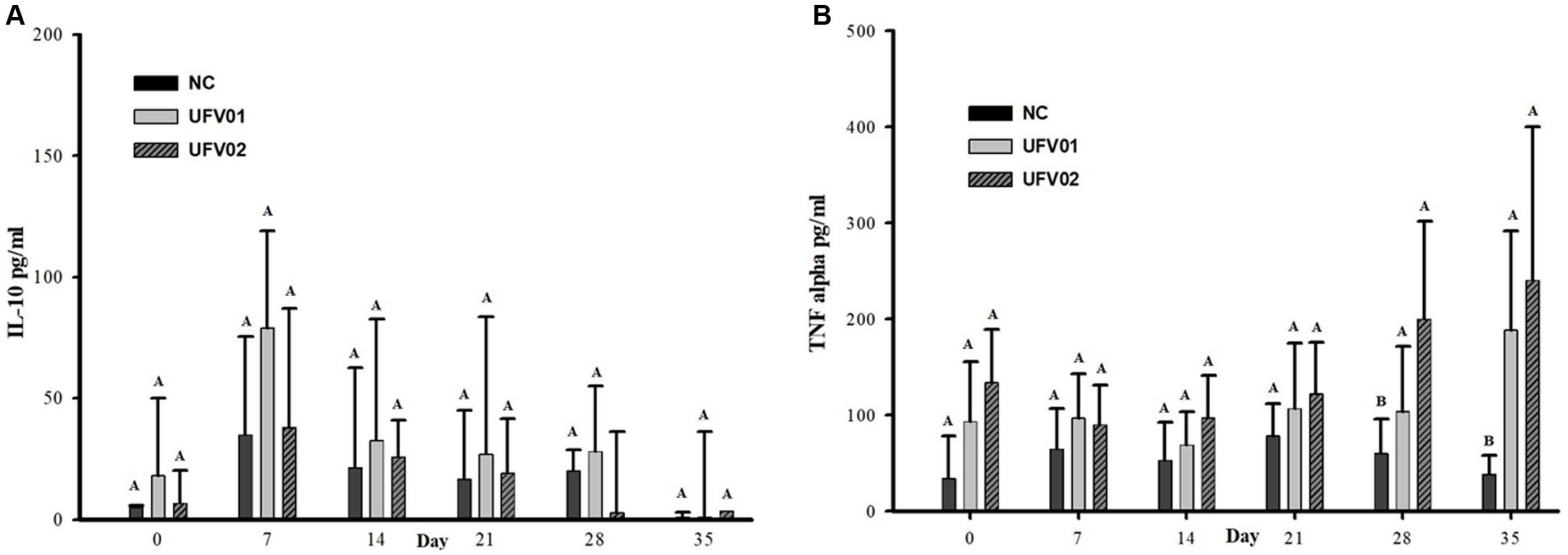
Dynamic graph of cytokine detection in the serum of swine experimentally inoculated with *Mycoplasma hyopneumoniae* with the UFV01 and UFV02 isolates. **(A)** IL-10 anti-inflammatory detections in pg./ml. **(B)** TNF-alpha proinflammatory cytokine detection in pg./mL. Different letters indicate a significant difference detected in the Tukey’s test (*p* < 0.05).

Cytokines IL-10, IFN-gamma, and TNF-alpha were investigated in serum samples using commercial kits. IFN-gamma was not detected in any of the animal sera at all time points, possibly indicating that its blood concentration was below the test’s detection range. Regarding cytokine IL-10, as shown in Figure 10A, there was no significant increase or decrease throughout the experimental dynamics.

TNF-alpha remained higher in the UFV01 and UFV02 groups compared to the NC group at 0, 7, 14, and 21 dpi, but without reaching statistical significance. Only at 28 and 35 dpi did the UFV01 and UFV02 groups show statistically significant differences compared to the NC group (Figure 10B).

### 3.6 Differential diagnosis

All animals tested negative for Influenza A virus, *Streptococcus suis* and *Glaesserella parasuis*. The UFV01 and UFV02 groups showed 87.5% (7/8) positivity for *Mycoplasma hyorhinis*. As for *Actinobacillus pleuropneumoniae*, the UFV01 group exhibited 25% positivity (animals H and K; 2/8). All three groups tested 100% (8/8 and 4/4) positive for *Pasteurella multocida*.

## 4 Discussion

The comparative genomic analysis of two strains of *M. hyopneumoniae* and their distinct aspects related to virulence and pathogenicity can be better evaluated when associated with a series of biological processes and characteristics in clinical signs in swine. The UFV01 and UFV02 genomes, isolated in Brazil, had distinct characteristics related to their genomes even though they are genetically related to each other and to other genomes deposited in the NCBI database. The UFV01 strain had fewer annotated genes than strain UFV02, as well as other related yet distinct genetic traits such as nitrogen metabolism, nucleosides and nucleotides, and respiration. It also exclusively presented the *KpnF* gene related to the efflux pump, and had more exclusive genetic clusters (singletons) in its genome. Interestingly, and possibly associated with its genetic characteristics, a higher cough frequency was recorded in the UFV01 group than in the UFV02 group. It also induced more severe macroscopic lung lesions and exclusively presented pleuritis. In addition, seroconversions were higher for both IgG and IgA for UFV01 than for UFV02. On the other hand, TNF-alpha progressively increased for both strains during the post-infection measurements.

The genome analysis of the UFV01 and UFV02 isolates reported genetic features similar to those of other strains of *M. hyopneumoniae*. The predicted virulence factors *mhp385, mhp271, MHP_RS03455, p102, p97, p216, MHP_RS00555,* and *mhp107* have been found in other studies (Zhang et al., 1995; Minion et al., 2004; Seymour et al., 2011; Bogema et al., 2012; Deutscher et al., 2012; Siqueira et al., 2013, 2014; Raymond and Djordjevic, 2015; Kamminga et al., 2020; Leal Zimmer et al., 2020; Pan et al., 2022), with their functions described as adhesin mechanisms allowing the bacteria to establish in the host cell (Zhang et al., 1995; Woolley et al., 2012; Siqueira et al., 2014; Bustamante-Marin and Ostrowski, 2017). The ST-123 assigned to both isolates seems to be an uncommon typing in Brazil, being only reported by Balestrin et al. (2019), which described only two isolates classified as ST-123. Furthermore, the ST-123 strain seems to be evolutionarily close to the ST-124 strain, differing by three pair bases on the *rpoB* gene allele.

Overall, 58–60% of the internal content (CDS) of *M. hyopneumoniae* was classified, standing according with (Tavares et al., 2022). The protein characterized as hypothetical can be attributed to the research timeline, specifically the inherent difficulties in isolating and sequencing these specific bacteria. The first reports of the whole genome sequences of this genus were in 2004–2005 with the 232 (Minion et al., 2004), 7,448 and J strains (Vasconcelos et al., 2005), and thus far only 24 isolates have been reported. Even with new references updating or adding new information on some strains (Darby et al., 1999) the literature provides little information about the genome’s features.

The UFV isolates presented the toxins *tlyC* (putative hemolysin), *PLDc_2* (PLD-like domain) and *hcnC* (hydrogen cyanide synthase HcnC). *PLDc_2* (PLD-like domain) was related to *Acinetobacter baumannii* and facilitates the invasion of the host cell (Stahl et al., 2015), and the toxin *hcnC* (hydrogen cyanide synthase) seems to be common in *Pseudomonas* spp., producing cyanide and acting lethally against Drosophila and nematodes (Darby et al., 1999; Broderick et al., 2008). The toxin *tlyC* was reported in other bacteria, such as *Rickettsia typhi* and *Leptospira*, showing no hemolytic action (Radulovic et al., 1999; Carvalho et al., 2009). However, *Brachyspira hyodysenteriae,* a bacterium related to enteric diseases in swine has the *tlyC* gene with hemolytic action (Joerling et al., 2020). In this case, the *tlyC* gene action of the UFV01 and UFV02 isolates needs more study to conclude on the hemolytic features. However, it is hypothesized that the isolates may have obtained the gene from *Brachyspira hyodysenteriae*, an organism of the digestive tract.

The only resistance gene was *KpnF* in UFV02. This gene belongs to the small multidrug resistance family (SMR) efflux pump-related genes, which show resistance to a broad range of drugs including a wide variety of antibiotics, antiseptics and disinfectants (Bay and Turner, 2009). This gene was first reported in *Klebsiella pneumoniae*, promoting antimicrobial resistance (Srinivasan and Rajamohan, 2013) and showing a large spectrum between the β-lactam family (Maurya et al., 2019). This gene was also reported in other *Enterobacteriaceae* such as *Salmonella* (Vilela et al., 2022), but never in *Mycoplasma*.

The genome comparison of the two isolates presented a similar size, with the UFV02 being larger than the others. In the BRIG analysis, the isolates of this study presented fewer gaps in comparison to the other assemblies, and the pan-genome analysis revealed a robust relationship between the core-genome comparison, except for the TB1 strain. In the region of variable cluster number, UFV01 and UFV02 seem to have the most clusters and singletons, being able to provide more information and explain the phenotypical results observed in the UFV01. In addition, the orthogroup analysis (Figure 3) showed three specific groups belonging only to the UFV isolates and the total separation from the NCBI strains in the dendrogram comparison.

Interestingly, UFV01 and UFV02 showed few differences in the subsystems identified in their genomes, being these differences in the subsystem: Energy and Precursor Metabolites Generation, Protein Synthesis, RNA Processing and Stress Response, Defense and Virulence. Despite the low differences between UFV01 and UFV02, these alterations between the strains, which may corroborate the differences presented in the symptoms and characteristics of the lesions presented. This complex interplay between host and pathogen requires the adaptation of bacterial virulence secretion systems and the innate immune recognition of conserved bacterial molecules (Haraga et al., 2008). Second (Rohmer et al., 2011), under certain circumstances, the metabolic capabilities of a pathogen may undergo changes as a result of functional loss due to mutations, which can confer an advantage within a specific environment. Additionally, it is conceivable that the loss of non-essential metabolic functions could enhance virulence by reducing the metabolic demands on pathways.

The control group did not present clinical signs compatible with enzootic pneumonia. The UFV01 group presented a numerically higher frequency of variation compared to the UFV02 group (Figure 5A). All three groups tested statistically differed from each other using the non-parametric Wilcoxon test *p* < 0.001 (Figure 5B). Garcia-Morante et al. (2022) highlight that the onset of coughing in experimentally infected pigs can be variable and intermittent, occurring between 1 and 3 weeks after infection. The immunopathological and environmental mechanisms leading to the formation of macroscopic lesions and, consequently, the manifestations of clinical disease are multifactorial. However, in experimental infections with virulent strains, it is described that the peak of the macroscopic lesion occurs at 28 dpi and decreases over time, eventually forming scar tissue (Vicca et al., 2003; Redondo et al., 2009; Garcia-Morante et al., 2017, 2022).

The group inoculated with the UFV01 strain exhibited 11.75% (+6.60) of lungs affected by lesions, the group inoculated with the UFV02 strain showed 3.125% (+1.55), and the negative control group showed 0% lesions at 35 dpi. The three groups differed statistically from each other. Almeida et al. (2020), in an experimental infection using the 232 strain (United States), found that the mean lesion area obtained in the lungs of animals at 28 dpi was 15.84%; this strain is considered to be highly virulent according to several studies (Leal Zimmer et al., 2020; Wu et al., 2022). Vicca et al. (2003), when comparing different isolates in an experimental infection, classified them as high, medium, and low pathogenicity, with the high virulence strains presenting an extent of the lesions in the lungs of inoculated pigs between 13.5% (±7.3) and 15.8% (±7.4), while the low pathogenicity isolates showed this indicator to be between 1.7 (±2.3) and 5.1 (±4.8). Therefore, analyzing the results and parameters from Almeida et al. (2020) and Vicca et al. (2003), the UFV01 isolate demonstrates the ability to cause a higher degree of macroscopic lung lesions than the UFV02 strain. Considering the parameters indicated in the aforementioned works, the UFV01 isolate presented high/medium virulence, while the UFV02 strain exhibited low pathogenicity.

As for the microscopic lesion scoring, the NC group did not present any type of lesion. The UFV01 and UFV02 groups showed bronchopneumonia and BALT, but with different frequencies. Additionally, only the UFV01 group presented cases of pleurisy, indicating secondary involvement. The UFV01 group showed 25% positivity for *Actinobacillus pleuropneumoniae*, 87.5% positivity for *Mycoplasma hyorhinis*, and 100% positivity for *Pasteurella multocida*. In contrast, the UFV02 group was positive for only *Mycoplasma hyorhinis* and *Pasteurella multocida*, without presenting cases of pleurisy. Positive animals for *Actinobacillus pleuropneumoniae* (H and K) in their lungs can be observed in Figure 6. During necropsy in both animals, pleural adhesions to the thoracic cavity can be observed, with more pronounced adhesion in animal K. Some Brazilian studies, such as Baraldi et al. (2019), Galdeano et al. (2019), and Petri et al. (2023), report a high prevalence of this agent in Brazilian production and its close association with *M. hyopneumoniae.* The presence of the *Actinobacillus pleuropneumoniae* together with *M. hyopneumoniae* tends to worsen bronchopneumonia lesions bronchopneumonia. The secondary involvement evidenced by the cases of pleurisy in the UFV01 group potentially occurred due to the fact that this isolate stimulated a higher degree of lesions, opening the door to secondary respiratory agents.

The mean scores of the microscopic lesions in the UFV01 group were 1.62 (bronchopneumonia), 1.5 (BALT hyperplasia) and 0.5 (pleuritis). The UFV02 group showed mean scores of 1.37 (bronchopneumonia), 1.12 (BALT hyperplasia) and 0.0 (pleuritis). Lymphocytic recruitment and its mitotic capacity in lung tissue leads to the described lesion known as BALT hyperplasia (Messier and Ross, 1991), which is considered a typical lesion caused by *M. hyopneumoniae*. On the other hand, bronchopneumonia and pleuritis lesions result from secondary bacterial involvement, including agents such as *Pasteurella multocida*, *Actinobacillus pleuropneumoniae*, *Streptococcus suis* and *Glaesserella parasuis* (De Conti et al., 2021).

Another important point evaluated was the seroconversion of animals through the enzyme-linked immunosorbent assay (ELISA). After the challenge, *M. hyopneumoniae* colonizes the respiratory tract and stimulates humoral immune responses, resulting in the production of IgG and IgA antibodies (Ding et al., 2021). In the study, seroconversion was not uniform. The UFV01 group started seroconversion at 21 dpi and achieved complete seroconversion at 35 dpi (100%), while the UFV02 group had only 50% seroconversion at 35 dpi. This result indicates that the host’s immune response against the UFV01 isolate was greater than against the UFV02 isolate, consequently leading to a more pronounced adaptive immune response to *M. hyopneumoniae*. From 21 dpi onwards, the UFV01 group showed statistically significant differences in antibody levels compared to the other groups (Figure 6A). In a study evaluating the seroconversion dynamics of animals challenged with the 232 strain (United States), seroconversion was found to start at 14 dpi, with the peak occurring at 35 dpi (Almeida et al., 2020).

Humoral responses after experimental infection show that specific serum IgG antibodies against *M. hyopneumoniae* are detected 3 to 4 weeks post-infection, reach their peak after 11 to 12 weeks, and then gradually decrease (Kobisch et al., 1993). After a booster infection, serum antibody titers clearly increase and then slowly decrease again (Kobisch et al., 1993). Interestingly, pigs infected with a highly virulent strain seem to seroconvert earlier than those infected with a low virulence strain (Meyns et al., 2004; Villarreal et al., 2011). This fact is confirmed when considering classifying the UFV01 isolate as high or medium virulence and the UFV02 isolate as low virulence, following the classification adopted by Vicca et al. (2003), as the seroconversion in the UFV01 group was higher and earlier compared to the UFV02 group.

IgA plays an important role in mucosal immunity against infectious pathogens. However, the molecular mechanism of IgA secretion in response to infection remains largely unknown, particularly in *Mycoplasma* spp. (Li et al., 2020). We assessed the level of IgA antibodies in the groups from BALF using a standardized method Mechler-dreibi et al. (2021), finding that 100% (8/8) of the animals in the UFV01 group tested positive for IgA detection, compared to only 50% (4/8) of the UFV02 group. The animals positive for serum IgG at 35 dpi were the same pigs that tested positive for IgA in BALF at 35 dpi (Figure 6B). It is believed that *M. hyopneumoniae* induces intense immune responses in mucosa, and long-lasting IgA may provide indispensable local immune protection for the against organism. However, few studies have examined the molecular mechanism by which *M. hyopneumoniae* promotes such strong mucosal immunity characterized by increased IgA levels (Li et al., 2020). The main strategy of respiratory tract mucosal immunity is forming a protective barrier to eliminate invasive respiratory pathogens and prevent active infection and colonization (Mechler-Dreibi et al., 2021). The ability of *M. hyopneumoniae* to adhere and multiply in the porcine respiratory epithelium is dependent on a complex set of adhesins and immunomodulatory strategies that allow *M. hyopneumoniae* to establish itself in the respiratory tract for long periods (Leal Zimmer et al., 2020).

The estimated bacterial quantification in the laryngeal swab increased over time (Figure 7A), although no statistical difference was detected between the UFV01 and UFV02 groups. Animals inoculated with the UFV01 strain showed environmental shedding of the bacteria, as 75% of the animals (6/8) tested positive in the qPCR of nasal swabs at 14 dpi, while the group inoculated with the UFV02 strain showed a detection rate of 37.5% (3/8) at 14 dpi (Figure 7A, Supplementary material S3). The qPCR analyzes for BALF and tissue in the UFV01 group showed 100% (8/8) positivity, while the UFV02 group showed 87.5% positivity (7/8). There was no statistical difference in bacterial load between the groups (Figure 7B; Supplementary material S3).

The production of pro-inflammatory cytokines has been associated with the development of characteristic lesions of EP (Muneta et al., 2008; Okamba et al., 2010; Jorge et al., 2014). TNF-alpha differed statistically between the groups at 28 and 35 dpi, with the animals inoculated with the UFV01 and UFV02 strains showed an increasing trend in this cytokine throughout the experimental period (Figure 8B). TNF-α is an important pro-inflammatory cytokine involved in the secretion of acute-phase proteins and is frequently detected in pigs infected with *M. hyopneumoniae* (Fourour et al., 2019). The consensus among the studies that have analyzed this cytokine is that it plays a role in the accumulation of inflammatory cells in lung tissue, contributing to lesion formation, although the exact timing of its peak expression and decrease has not yet been fully elucidated (Choi et al., 2006; Lorenzo et al., 2006; Redondo et al., 2009; Almeida et al., 2020).

Therefore, UFV01 and UFV02 presented genetic orthology characteristics that differ from the other sequences deposited in international banks, in terms of different ortho groups and similar phylogenomics. In contrast, the functional annotations demonstrate that the two strains are genetically different, as demonstrated in the symptoms presented by the animals. However, the limitations on understanding genetic functions as a whole require further studies. Two important points can be observed between the groups. In the UFV01 group, the animal with the highest degree of lung involvement died. Hence, this group had a mortality rate of 12.5%. On the other hand, the UFV02 group had an animal that, despite being inoculated similarly to the others, did not develop EP and tested negative for the agent throughout the study. Both pieces of information support the presence of differences in virulence between the groups, with UFV01 being more pathogenic. Despite the data presented in our study, further research is needed to deepen our understanding of the genetic differences and their phenotypic presentations.

The UFV01 and UFV02 strains are capable of producing clinical disease. After a thorough analysis of the discussed data, it is possible to infer that the UFV01 strain represents a more pathogenic isolate compared to the UFV02 strain. However, further studies are necessary to elucidate the genetic differences and their phenotypic presentations. New research can stem from our findings, such as the development of vaccines, antimicrobial assessments, diagnostic kits and improvements in control protocols.

## Data availability statement

The datasets presented in this study can be found in online repositories. The names of the repository/repositories and accession number(s) can be found in the article/Supplementary material.

## Ethics statement

The animal study was approved by Committee on Ethics in the Use of Animals (CEUA). The study was conducted in accordance with the local legislation and institutional requirements.

## Author contributions

LT: Writing – original draft, Writing – review & editing, Data curation, Investigation. LS: Investigation, Writing – original draft, Conceptualization, Methodology, Validation. CP: Conceptualization, Methodology, Formal analysis, Writing – original draft, Writing – review & editing. RP: Formal analysis, Methodology, Writing – original draft, Data curation, Writing – review & editing. GB: Methodology, Conceptualization, Resources, Writing – original draft. RY: Methodology, Data curation, Formal analysis, Writing – original draft. KJ: Data curation, Formal analysis, Methodology, Writing – review & editing. FM: Formal analysis, Methodology, Investigation, Writing – review & editing. CD: Formal analysis, Methodology, Writing – original draft. VC: Formal analysis, Methodology, Validation, Writing – original draft. CM: Formal analysis, Methodology, Data curation, Writing – original draft. FP: Formal analysis, Methodology, Writing – original draft. LO: Methodology, Conceptualization, Data curation, Supervision, Writing – original draft. MM: Conceptualization, Supervision, Writing – original draft. AS-J: Conceptualization, Supervision, Writing – original draft, Funding acquisition, Methodology, Project administration, Resources, Writing – review & editing.

## Footnotes

^1^http://www.mgc.ac.cn/VFs/main.htm
