# Supplementary material for: A genetic and virulence characterization of Brazilian strains of Mycoplasma hyopneumoniae
Source: Front Microbiol. 2023 Nov 22;14:1280588. doi: 10.3389/fmicb.2023.1280588 (PMC10702778; doi:10.3389/fmicb.2023.1280588)
Supplement: Supplementary file 1 [file Table_1.docx]

| **Name** | **GenBank accession number** | **Genome size (Mb)** | **% G + C** | **CDS** | **Open access GenBank** | **Year of isolation** | | **Country of origin** | **Virulence** |
| --- | --- | --- | --- | --- | --- | --- | --- | --- | --- |
| [UFV02](https://www.ncbi.nlm.nih.gov/genome/190?genome_assembly_id=1818604) | [PRJNA542605](https://www.ncbi.nlm.nih.gov/bioproject/PRJNA542605) | 0.959419 | 28,5 | [709](https://www.ncbi.nlm.nih.gov/genome/browse/#!/proteins/190/1818604%7CMesomycoplasma%20hyopneumoniae/) | 2022 | 2017 | | Brazil | +^*^ |
| [UFV01](https://www.ncbi.nlm.nih.gov/genome/190?genome_assembly_id=1818603) | [PRJNA542605](https://www.ncbi.nlm.nih.gov/bioproject/PRJNA542605) | 0.909816 | 28.4 | [674](https://www.ncbi.nlm.nih.gov/genome/browse/#!/proteins/190/1818603%7CMesomycoplasma%20hyopneumoniae/) | 2022 | 2017 | | Brazil | +^*^ |
| [TB1](https://www.ncbi.nlm.nih.gov/genome/190?genome_assembly_id=322533) | [PRJNA378902](https://www.ncbi.nlm.nih.gov/bioproject/PRJNA378902) | 0.909064 | 28.7 | [707](https://www.ncbi.nlm.nih.gov/genome/browse/#!/proteins/190/322533%7CMesomycoplasma%20hyopneumoniae/) | 2017 | 2016 | | China | + |
| [NCTC10127](https://www.ncbi.nlm.nih.gov/genome/190?genome_assembly_id=445253) | [PRJEB6403](https://www.ncbi.nlm.nih.gov/bioproject/PRJEB6403) | 0.960532 | 28.53 | [738](https://www.ncbi.nlm.nih.gov/genome/browse/#!/proteins/190/445253%7CMesomycoplasma%20hyopneumoniae/) | 2019 | 2016 | | Suíça | ND |
| [MHP709](https://www.ncbi.nlm.nih.gov/genome/190?genome_assembly_id=753471) | [PRJNA478906](https://www.ncbi.nlm.nih.gov/bioproject/PRJNA478906) | 0.875793 | 28.6 | [629](https://www.ncbi.nlm.nih.gov/genome/browse/#!/proteins/190/753471%7CMesomycoplasma%20hyopneumoniae/) | 2020 | 2016 | | France | ND |
| [MHP699](https://www.ncbi.nlm.nih.gov/genome/190?genome_assembly_id=753469) | [PRJNA478906](https://www.ncbi.nlm.nih.gov/bioproject/PRJNA478906) | 0.918878 | 28.5 | [641](https://www.ncbi.nlm.nih.gov/genome/browse/#!/proteins/190/753469%7CMesomycoplasma%20hyopneumoniae/) | 2020 | 2016 | | France | ND |
| [MHP696](https://www.ncbi.nlm.nih.gov/genome/190?genome_assembly_id=753468) | [PRJNA478906](https://www.ncbi.nlm.nih.gov/bioproject/PRJNA478906) | 0.874552 | 28.6 | [630](https://www.ncbi.nlm.nih.gov/genome/browse/#!/proteins/190/753468%7CMesomycoplasma%20hyopneumoniae/) | 2020 | 2016 | | France | ND |
| [MHP694](https://www.ncbi.nlm.nih.gov/genome/190?genome_assembly_id=753470) | [PRJNA478906](https://www.ncbi.nlm.nih.gov/bioproject/PRJNA478906) | 0.867508 | 28.6 | [621](https://www.ncbi.nlm.nih.gov/genome/browse/#!/proteins/190/753470%7CMesomycoplasma%20hyopneumoniae/) | 2020 | 2016 | | France | ND |
| [MHP691](https://www.ncbi.nlm.nih.gov/genome/190?genome_assembly_id=753467) | [PRJNA478906](https://www.ncbi.nlm.nih.gov/bioproject/PRJNA478906) | 0.897976 | 28.5 | [646](https://www.ncbi.nlm.nih.gov/genome/browse/#!/proteins/190/753467%7CMesomycoplasma%20hyopneumoniae/) | 2020 | 2016 | | France | ND |
| [MHP682](https://www.ncbi.nlm.nih.gov/genome/190?genome_assembly_id=753474) | [PRJNA478906](https://www.ncbi.nlm.nih.gov/bioproject/PRJNA478906) | 0.905968 | 28.6 | [646](https://www.ncbi.nlm.nih.gov/genome/browse/#!/proteins/190/753474%7CMesomycoplasma%20hyopneumoniae/) | 2020 | 2016 | | France | ND |
| [MHP679](https://www.ncbi.nlm.nih.gov/genome/190?genome_assembly_id=753475) | [PRJNA478906](https://www.ncbi.nlm.nih.gov/bioproject/PRJNA478906) | 0.901315 | 28.6 | [665](https://www.ncbi.nlm.nih.gov/genome/browse/#!/proteins/190/753475%7CMesomycoplasma%20hyopneumoniae/) | 2020 | 2016 | | France | ND |
| [MHP653](https://www.ncbi.nlm.nih.gov/genome/190?genome_assembly_id=753473) | [PRJNA478906](https://www.ncbi.nlm.nih.gov/bioproject/PRJNA478906) | 0.892502 | 28.5 | [643](https://www.ncbi.nlm.nih.gov/genome/browse/#!/proteins/190/753473%7CMesomycoplasma%20hyopneumoniae/) | 2020 | 2016 | | France | ND |
| [MHP650](https://www.ncbi.nlm.nih.gov/genome/190?genome_assembly_id=753472) | [PRJNA478906](https://www.ncbi.nlm.nih.gov/bioproject/PRJNA478906) | 0.903416 | 28.6 | [654](https://www.ncbi.nlm.nih.gov/genome/browse/#!/proteins/190/753472%7CMesomycoplasma%20hyopneumoniae/) | 2020 | 2016 | | France | ND |
| [LE](https://www.ncbi.nlm.nih.gov/genome/190?genome_assembly_id=1761537) | [PRJNA741719](https://www.ncbi.nlm.nih.gov/bioproject/PRJNA741719) | 0.920587 | 28.5 | [693](https://www.ncbi.nlm.nih.gov/genome/browse/#!/proteins/190/1761537%7CMesomycoplasma%20hyopneumoniae/) | 2022 | 2012 | | China | ND |
| [KM014](https://www.ncbi.nlm.nih.gov/genome/190?genome_assembly_id=325793) | [PRJNA396462](https://www.ncbi.nlm.nih.gov/bioproject/PRJNA396462) | 0.964503 | 28.4 | [900](https://www.ncbi.nlm.nih.gov/genome/browse/#!/proteins/190/325793%7CMesomycoplasma%20hyopneumoniae/) | 2017 | 2014 | | South Korea | + |
| [J](https://www.ncbi.nlm.nih.gov/genome/190?genome_assembly_id=299966) | [PRJNA10675](https://www.ncbi.nlm.nih.gov/bioproject/PRJNA10675) | 0.897405 | 28.5 | [672](https://www.ncbi.nlm.nih.gov/genome/browse/#!/proteins/190/299966%7CMesomycoplasma%20hyopneumoniae%20J/) | 2005 | 1957 | UK | | - |
| [F7.2C](https://www.ncbi.nlm.nih.gov/genome/190?genome_assembly_id=654241) | [PRJNA506155](https://www.ncbi.nlm.nih.gov/bioproject/PRJNA506155) | 0.894983 | 28.6 | [676](https://www.ncbi.nlm.nih.gov/genome/browse/#!/proteins/190/654241%7CMesomycoplasma%20hyopneumoniae/) | 2019 | 2000 | | Bélgica | + |
| [ES-2L](https://www.ncbi.nlm.nih.gov/genome/190?genome_assembly_id=917860) | [PRJNA576551](https://www.ncbi.nlm.nih.gov/bioproject/PRJNA576551) | 0.918900 | 28.5 | [686](https://www.ncbi.nlm.nih.gov/genome/browse/#!/proteins/190/917860%7CMesomycoplasma%20hyopneumoniae/) | 2020 | 2019 | | China | + |
| [ES-2](https://www.ncbi.nlm.nih.gov/genome/190?genome_assembly_id=490696) | [PRJNA530402](https://www.ncbi.nlm.nih.gov/bioproject/PRJNA530402) | 0.956514 | 28.4 | [718](https://www.ncbi.nlm.nih.gov/genome/browse/#!/proteins/190/490696%7CMesomycoplasma%20hyopneumoniae/) | 2019 | 2017 | | China | ND |
| [98](https://www.ncbi.nlm.nih.gov/genome/190?genome_assembly_id=919079) | [PRJNA593525](https://www.ncbi.nlm.nih.gov/bioproject/PRJNA593525) | 0.880620 | 28.6 | [635](https://www.ncbi.nlm.nih.gov/genome/browse/#!/proteins/190/919079%7CMesomycoplasma%20hyopneumoniae/) | 2020 | 2014 | | Netherlands | ND |
| [7448](https://www.ncbi.nlm.nih.gov/genome/190?genome_assembly_id=299967) | [PRJNA10639](https://www.ncbi.nlm.nih.gov/bioproject/PRJNA10639) | 0.920079 | 28.5 | [689](https://www.ncbi.nlm.nih.gov/genome/browse/#!/proteins/190/299967%7CMesomycoplasma%20hyopneumoniae%207448/) | 2005 | 2005 | | Brazil | + |
| [7422](https://www.ncbi.nlm.nih.gov/genome/190?genome_assembly_id=299969) | [PRJNA47327](https://www.ncbi.nlm.nih.gov/bioproject/PRJNA47327) | 0.898495 | 28.5 | [673](https://www.ncbi.nlm.nih.gov/genome/browse/#!/proteins/190/299969%7CMesomycoplasma%20hyopneumoniae%207422/) | 2013 | 2013 | | Brazil | + |
| [232](https://www.ncbi.nlm.nih.gov/genome/190?genome_assembly_id=299968) | [PRJNA13120](https://www.ncbi.nlm.nih.gov/bioproject/PRJNA13120) | 0.892758 | 28.6 | [674](https://www.ncbi.nlm.nih.gov/genome/browse/#!/proteins/190/299968%7CMesomycoplasma%20hyopneumoniae%20232/) | 2004 | 2004 | | USA | + |
| [168-L](https://www.ncbi.nlm.nih.gov/genome/190?genome_assembly_id=165760) | [PRJNA76853](https://www.ncbi.nlm.nih.gov/bioproject/PRJNA76853) | 0.921093 | 28.5 | [697](https://www.ncbi.nlm.nih.gov/genome/browse/#!/proteins/190/165760%7CMesomycoplasma%20hyopneumoniae%20168-L/) | 2013 | 1974 | | China | - |
| [168](https://www.ncbi.nlm.nih.gov/genome/190?genome_assembly_id=165759) | [PRJNA53881](https://www.ncbi.nlm.nih.gov/bioproject/PRJNA53881) | 0.925576 | 28.5 | [694](https://www.ncbi.nlm.nih.gov/genome/browse/#!/proteins/190/165759%7CMesomycoplasma%20hyopneumoniae%20168/) | 2010 | 1974 | | China | + |
| [11](https://www.ncbi.nlm.nih.gov/genome/190?genome_assembly_id=321540) | [PRJNA378403](https://www.ncbi.nlm.nih.gov/bioproject/PRJNA378403) | 0.898117 | 28.7 | [670](https://www.ncbi.nlm.nih.gov/genome/browse/#!/proteins/190/321540%7CMesomycoplasma%20hyopneumoniae/) | 2017 | 2012 | | Netherlands | + |

26 sequenced genomes deposited in the GenBank database. Pathogenic (+); non-pathogenic (-); not described (ND).
