# Supplementary material for: A genetic and virulence characterization of Brazilian strains of Mycoplasma hyopneumoniae
Source: Front Microbiol. 2023 Nov 22;14:1280588. doi: 10.3389/fmicb.2023.1280588 (PMC10702778; doi:10.3389/fmicb.2023.1280588)
Supplement: Supplementary file 2 [file Table_2.DOCX]

Table S2: List of primers and probes for each of the bacterial and viral pathogens tested in qPCR.

| Pathogen | Gene | Primers Sequence (5'-3') | | Amplicon (pb) | probe (5'-3') | Reference |
| --- | --- | --- | --- | --- | --- | --- |
|  |  |  | |  |  |  |
| *Mycoplasma hyopneumoniae* | adesina p102 | F | 5′-TAAGGGTCAAAGTCAAAGTC-3′ | 150 | 5′- FAM-AACCAGTTTCCACTTCATCGCC-§BHQ2−3’ | Fourour et al. (2018) |
|  |  | R | 5′-AAATTAAAAGCTGTTCAAATGC-3′ |  |  |  |
| *Actinobacillus pleuropneumoniae* | omlA | F | 5'-AGTGCTTACCGCATGTAGTGGC-3' | 153 | 5′-FAM-CGATGAACCCGATGAGCCGCC-3′-TAMRA | Goecke et al. (2020) |
|  |  | R | 5'-TTGGTGCGGACATATCAACCTTA-3' |  |  |  |
| *Pasteurella multocida* | kmt1 | F | 5'-GGGCTTGTCGGTAGTCTTT -3' | 148 | 5'-FAM-CGGCGCAACTGATTGGACGTTATT-TAMRA-3' | Sunaga et al. (2019) |
|  |  | R | 5'-CGGCAAATAACAATAAGCTGAGTA-3' |  |  |  |
| *Glaesserella parasuis* | infB | F | 5'-CGACTTACTTGAAGCCATTCTTCTT-3' | 75 | 5'-FAM-ATCGGAAGTATTAGAATTAAGTGC-TAMRA-3' | Han (2020) |
|  |  | R | 5'-CCGCTTGCCATACCCTCTT-3' |  |  |  |
| *Streptococcus suis* | cps2J | F | 5′-GGTTACTTGCTACTTTTGATGGAAATT-3′ | 85 | 5′-FAM-TCAAGAATCTGAGCTGCAAAAGTGTCAAATTGA-TAMRA-3′ | Bonifait et al. (2014) |
|  |  | R | 5′-CGCACCTCTTTTATCTCTTCCAA-3′ |  |  |  |
| *Mycoplasma*  *hyorhinis* | p37 | F | 5′-TTCTATTTTCATCTATATTTTCGC-3′ | 101 | 5′- TXR b -CATCCTCTTGCTTGACTACTCCTG -BHQ2−3’ | Fourour et al. (2018) |
|  |  | R | 5′-TCATTGACCTTGACTAACTG -3′ |  |  |  |
| *Influenza A virus* | Matrix gene | F | 5′-  CTTCTAACCGAGGTCGAAACG  -3′ | 244 | N.A. | Fouchier et al. (2000) |
|  |  | R | 5′-  AGGGCATTTTGGACAAATCGTCTA  -3′ |  | N.A. |  |

N.A. non-applicable.
