# Supplementary material for: A genetic and virulence characterization of Brazilian strains of Mycoplasma hyopneumoniae
Source: Front Microbiol. 2023 Nov 22;14:1280588. doi: 10.3389/fmicb.2023.1280588 (PMC10702778; doi:10.3389/fmicb.2023.1280588)
Supplement: Supplementary file 3 [file Table_3.DOCX]

Table S3: Detection of *Mycoplasma hyopneumoniae* DNA fragment copies in laryngeal swab, tracheobronchial lavage, and lesioned tissue of pigs experimentally inoculated with UFV01 and UFV02 isolates.

|  | **Laryngeal swab** | | | | | | | | **LTB** | **Tissue** |
| --- | --- | --- | --- | --- | --- | --- | --- | --- | --- | --- |
| **Groups** | **ID** | **-7** | **0** | **07 dpi** | **14 dpi** | **21 dpi** | **28 dpi** | **35 dpi** | **35 dpi** | **35 dpi** |
|  | **A** | **-** | **-** | **-** | **-** | **-** | **-** | **-** | **-** | **-** |
|  | **B** | **-** | **-** | **-** | **-** | **-** | **-** | **-** | **-** | **-** |
|  | **C** | **-** | **-** | **-** | **-** | **-** | **-** | **-** | **-** | **-** |
| **NC** | **D** | **-** | **-** | **-** | **-** | **-** | **-** | **-** | **-** | **-** |
|  | **Average** | **0** | **0** | **0** | **0** | **0** | **0** | **0** | **0** | **0** |
|  | **% Positives** | **0%** | **0%** | **0%** | **0%** | **0%** | **0%** | **0%** | **0%** | **0%** |
|  | **X** | **-** | **-** | **-** | 2.29x10^3^ | 1.09x10^2^ | 3.94x10^3^ | 0.00 | 1.32x10^4^ | 1.61x10^4^ |
|  | **F** | **-** | **-** | **-** | 2.72x10^2^ | 1.22x10^4^ | 3.26x10^3^ | 4.42x10^2^ | 7.76x10^6^ | 1.35x10^7^ |
|  | **G** | **-** | **-** | **-** | 4.81x10^2^ | 1.70x10^3^ | 1.80x10^3^ | 5.79x10^4^ | 2.32x10^6^ | 8.95x10^6^ |
|  | **H** | **-** | **-** | **-** | **-** | 3.77x10^2^ | 5.34x10^3^ | 1.25x10^4^ | 6.77x10^4^ | 1.14x10^7^ |
|  | **I** | **-** | **-** | **-** | **-** | **-** | 5.71x10^1^ | 2.87x10^4^ | 1.89x10^5^ | 1.41x10^6^ |
|  | **J** | **-** | **-** | **-** | 1.38x10^2^ | 2.59x10^2^ | 4.65x10^2^ | 7.37x10^2^ | 2.14x10^5^ | 7.98x10^3^ |
|  | **K** | **-** | **-** | **-** | 1.03x10^4^ | 1.05x10^3^ | 5.35x10^2^ | 4.82x10^3^ | 5.01x10^5^ | 2.14x10^5^ |
| **UFV01** | **L** | **-** | **-** | **-** | 1.05x10^2^ | 4.36x10^2^ | 1.07x10^3^ | 2.25x10^4^ | 6.16x10^5^ | 3.85x10^6^ |
|  | **Average** | **0** | **0** | **0** | **3.76x10^2^** | **4.36x10^2^** | **1.44x10^3^** | **1.25x10^4^** | **3.57x10^5^** | **2.63x10^6^** |
|  | **% Positives** | **0%** | **0%** | **0%** | **75%** | **87.50%** | **100%** | **87.50%** | **100%** | **100%** |
|  | **M** | **-** | **-** | **-** | **-** | 1.47x10^4^ | 4.20x10^3^ | 4.40x10^3^ | 2.08x10^6^ | 6.31x10^6^ |
|  | **N** | **-** | **-** | **-** | 6.27x10^2^ | 1.46x10^2^ | 4.63x10^4^ | 8.53x10^3^ | 6.32x10^6^ | 6.11x10^5^ |
|  | **O** | **-** | **-** | **-** | **-** | - | 4.45x10^2^ | 2.42x10^3^ | 8.40x10^5^ | 6.91x10^6^ |
|  | **P** | **-** | **-** | **-** | **-** | 1.50x10^3^ | 7.82x10^2^ | 2.43x10^3^ | 1.89x10^6^ | 3.92x10^6^ |
|  | **Q** | **-** | **-** | **-** | 1.82x10^2^ | 1.66x10^2^ | 4.72x10^5^ | 3.00x10^5^ | 1.27x10^7^ | 3.16x10^6^ |
|  | **R** | **-** | **-** | **-** | 1.36x10^2^ | 5.85x10^1^ | 7.59x10^2^ | 1.17x10^3^ | 1.09x10^7^ | 8.20x10^6^ |
|  | **S** | **-** | **-** | **-** | **-** | 9.74x10^3^ | 1.44x10^3^ | 2.50x10^3^ | 1.16x10^7^ | 2.39x10^5^ |
| **UFV02** | **T** | **-** | **-** | **-** | **-** | **-** | **-** | **-** | **-** | **-** |
|  | **Average** | **0** | **0** | **0** | **1.82x10^2^** | **8.33x10^2^** | **1.44x10^3^** | **2.50x10^3^** | **6.32x10^6^** | **3.92x10^6^** |
|  | **% Positives** | **0%** | **0%** | **0%** | **37.50%** | **75%** | **87.50%** | **87.50%** | **87.50%** | **87.50%** |

Negative (-), Positive (+).

Table 02: Efficiency of diluted standard curves for qPCR analyses.

| Fluor | Eficiência % | Slope | Y-Intercept | R^2^ |
| --- | --- | --- | --- | --- |
| FAM | 101,73 | -3,281 | 42,733 | 1,000 |
| FAM | 101,22 | -3,293 | 42,596 | 0,997 |
| FAM | 98,62 | -3,355 | 43,682 | 1,000 |
| FAM | 99,22 | -3,341 | 43,858 | 0,993 |
| FAM | 101,46 | -3,287 | 43,020 | 0,994 |
| FAM | 99,96 | -3,323 | 43,631 | 0,998 |
